# Analysis of XMRV integration sites from human prostate cancer tissues suggests PCR contamination rather than genuine human infection

**DOI:** 10.1186/1742-4690-8-13

**Published:** 2011-02-25

**Authors:** Jeremy A Garson, Paul Kellam, Greg J Towers

**Affiliations:** 1 MRC Centre for Medical Molecular Virology, Division of Infection and Immunity, University College London, 46 Cleveland St, London W1T 4JF, UK; 2 Wellcome Trust Sanger Institute, Hinxton, Cambridge, CB10 1SA, UK

## Abstract

XMRV is a gammaretrovirus associated in some studies with human prostate cancer and chronic fatigue syndrome. Central to the hypothesis of XMRV as a human pathogen is the description of integration sites in DNA from prostate tumour tissues. Here we demonstrate that 2 of 14 patient-derived sites are identical to sites cloned in the same laboratory from experimentally infected DU145 cells. Identical integration sites have never previously been described in any retrovirus infection. We propose that the patient-derived sites are the result of PCR contamination. This observation further undermines the notion that XMRV is a genuine human pathogen.

## Introduction

XMRV was originally described in 2006 in the tumour tissue of patients with a familial form of prostate cancer associated with mutations that impair the function of the antiviral defence protein RNase L [1]. Three independent groups subsequently reported the presence of XMRV in a significant proportion of prostate cancers, but the linkage to polymorphisms of the RNase L gene was not confirmed. In contrast, at least seven other studies have reported an inability to detect, or extremely low prevalence of, XMRV in prostate cancer despite using highly sensitive PCR-based assays.

Immunohistological, *in situ*-hybridisation and serological studies have also been inconsistent in their findings. Some studies [1,2], using immunostaining and/or FISH, detected XMRV in a small percentage of stromal cells but not in tumour cells, whereas others using similar techniques reported XMRV predominantly in tumour cells rather than stromal cells. In a recent study, Aloia and colleagues [3] employed HPLC purified proteins to raise the antisera used for immunostaining, and were unable to find any trace of XMRV at all in nearly 800 prostate tumours analysed. They suggested that the positive immunostaining described in earlier studies may have been due to the use of non-specific antisera exhibiting cross-reactivity with human cellular proteins [3].

Similar controversy surrounds claims of an association between XMRV and chronic fatigue syndrome (CFS). In a highly publicised study, Lombardi and colleagues detected XMRV in 67% of CFS patients and 3.7% of healthy controls by nested PCR [4]. Since Lombardi's initial publication, there have been numerous attempts by other groups in several countries to confirm the linkage between XMRV infection and CFS; but as yet none have succeeded. Curiously, one study described PCR detection of a second MLV (modified polytropic MLV), but not XMRV itself, in the blood of some CFS patients [5]. XMRV has also been sought in a variety of other diseases including amyotrophic lateral sclerosis, multiple sclerosis, autism, immunosuppression, rheumatoid arthritis, fibromyalgia and paediatric idiopathic disease; but all with negative results.

A number of recent publications have attempted to explain these confusing and highly contradictory reports by calling attention to the significant risk of false positive XMRV results due to laboratory contamination, and to PCR contamination in particular. The considerable potential for false positives arising from minute traces of murine DNA contaminating test samples or reagents has been clearly demonstrated as has the risk of erroneous results due to contamination from human tumour cell lines infected with XMRV (e.g. 22Rv1) or other xenotropic MLVs acquired by xenografting in mice [6].

## Integration of XMRV into human chromosomes

Central to the hypothesis that XMRV is a genuine human pathogen is the observation that it integrates into the chromosomal DNA of prostate tumour tissues [7,8]. Such integration of the cDNA copy of genomic viral RNA to form the provirus is essential for retroviruses to establish productive infection. Given the importance of this observation, we sought to examine the authenticity of the XMRV integration sites that have been reported to date.

**Figure 1:**
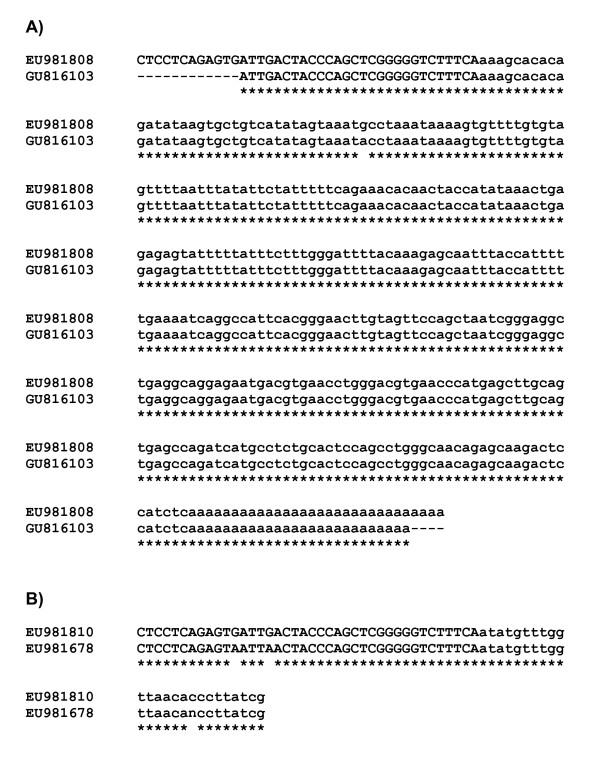
**Nucleotide alignments of XMRV integration site sequences derived from patients' prostate cancer tissues and the experimentally infected human tumour cell line DU145**. Panel (A) shows the alignment of sequence EU981808 (patient 122-derived) and sequence GU816103 (DU145 cell line-derived). Panel (B) shows the alignment of sequence EU981810 (patient VP268-derived) and sequence EU981678 (DU145 cell line-derived). The initial 169 nt segment of sequence EU981678 is not shown as it includes a repeat within the XMRV sequence which is not covered by the much shorter EU981810 sequence and is therefore redundant for purposes of alignment. Upper case letters represent the XMRV LTR sequence and lower case letters represent the flanking human chromosomal sequence. Note that the viral 3' ends terminate with a conserved CA dinucleotide.

The only research group describing patient-derived XMRV integration sites provides sequence data from 14 XMRV integration sites cloned from the prostatic tumour tissues of 9 patients [GenBank: EU981800 to GenBank: EU981813] [7,8]. Nucleotide BLAST searches using each of the 14 integration site sequences against the GenBank nr database revealed that 2 of the 14 integration sites [GenBank: EU981808 and GenBank: EU981810], obtained from two different patients, were identical to XMRV integration sites [GenBank: GU816103 and GenBank: EU981678] respectively, which were cloned from the experimentally infected human tumour cell line, DU145 [8,9] in the same laboratories (Figure 1A and 1B). Two mismatched nucleotides were noted in the LTR region between EU981810 and EU981678 (Figure 1B). These errors are possibly the result of somatic mutation in the cell line during its replication or the result of PCR error during amplification. PCR error is not unlikely given the three step nested PCR protocol and the non-proofreading enzyme (Taq2000) used in the amplification of this integration site (EU981810) from patient tissue [7].

## Discussion

Current knowledge based on the analysis of several thousand retroviral integration sites suggests that target site selection is not primarily sequence-specific, although different classes of retrovirus exhibit distinct genome location biases [10]. HIV-1 for example appears to favour integration into transcription units whereas MLV tends to integrate near transcription start sites and CpG islands. Both have a preference for gene dense regions. Analysis of several hundred XMRV integration site sequences [8,9] has revealed a preference for transcription start sites, CpG islands, DNase-hypersensitive sites and gene-dense regions as is typical for an MLV. Although primary DNA sequence is not regarded as a dominant factor in determining target site specificity, a weak palindromic consensus sequence for XMRV integration sites (namely, 5'-CTVB where V is A, C or G and B is C, G or T) has been identified [9]. With the exception of a single early publication on avian sarcoma-leukosis virus, which was refuted by later work [10], sequencing studies of thousands of retroviral integration sites have to our knowledge never identified exactly the same site twice. It therefore appears very unlikely that the sites illustrated in Figure 1 are the result of independent integrations into identical genomic locations in a prostate tumour *in vivo *and an experimentally infected cell line *in vitro*, on two separate occasions.

We consider PCR based contamination to be the most likely explanation for the identification of identical integration sites from the patients' prostate tumour tissues and from the DU145 cells experimentally infected with XMRV. It is noteworthy that the prostate tumour tissue sites and the DU145 cell sites were cloned by the same research group in the same laboratories [7-9] and that the GU816103 sequence was derived from a clonally amplified cell line [9]. The propensity for PCR contamination is increased due to the unusual technique used for cloning the prostate tissue-derived integration sites which involved an extraordinary degree of PCR amplification with 80 preliminary amplification cycles followed by nested PCR consisting of 29 cycles and then an additional 18 cycles [8]. PCR tubes were opened during the procedure for the addition of fresh DNA polymerase after 40 cycles. Using such a technique would entail a significant risk of direct or indirect contamination from experimentally infected DU145 cells, cellular DNA, plasmids or PCR products that had been handled in the same environment. No negative controls were mentioned in the published method [8]. Although it remains theoretically possible that contamination occurred in the reverse direction, i.e. from the patient-derived tumour tissue to the DU145 cell line, we consider this to be exceedingly unlikely.

Whilst it is conceivable that the other 12 integration sites apparently derived from prostatic tumour tissues [7,8] are genuine patient-derived sequences, we suspect that some or all of them may also be the result of contamination with DNA from experimentally infected DU145 cells. It is striking that there have been no independent reports of patient-derived XMRV integration sites nor have there been any descriptions of polytropic or modified polytropic MLV integration sites in human samples despite the apparent detection of these viruses in CFS patients [5]. In conclusion, we believe that our findings undermine a central component of the evidence for XMRV being a human pathogen.

## List of abbreviations

CFS: chronic fatigue syndrome; FISH: fluorescence in situ hybridization; HIV-1: human immunodeficiency virus type 1; HPLC: high performance liquid chromatography; LTR: long terminal repeat; MLV: murine leukaemia virus; PCR: polymerase chain reaction; XMRV: Xenotropic murine leukaemia virus-related virus.

## Competing interests

The authors declare that they have no competing interests.

## Authors' contributions

JAG conceived the study and performed the research. JAG, GJT and PK interpreted the data and wrote the paper. All authors read and approved the final manuscript.

## References

[B1] Urisman A Molinaro RJ Fischer N Plummer SJ Casey G Klein EA Malathi K Magi-Galluzzi C Tubbs RR Ganem D Silverman RH DeRisi JL Identification of a novel Gammaretrovirus in prostate tumors of patients homozygous for R462Q RNASEL variant PLoS Pathog 2006 2 e25 10.1371/journal.ppat.0020025 16609730 PMC1434790

[B2] Arnold RS Makarova NV Osunkoya AO Suppiah S Scott TA Johnson NA Bhosle SM Liotta D Hunter E Marshall FF Ly H Molinaro RJ Blackwell JL Petros JA XMRV infection in patients with prostate cancer: novel serologic assay and correlation with PCR and FISH Urology 2010 75 755 761 10.1016/j.urology.2010.01.038 20371060

[B3] Aloia AL Sfanos KS Isaacs WB Zheng Q Maldarelli F De Marzo AM Rein A XMRV: a new virus in prostate cancer? Cancer Res 2010 70 10028 10033 10.1158/0008-5472.CAN-10-2837 20966126 PMC3005136

[B4] Lombardi VC Ruscetti FW Das Gupta J Pfost MA Hagen KS Peterson DL Ruscetti SK Bagni RK Petrow-Sadowski C Gold B Dean M Silverman RH Mikovits JA Detection of an infectious retrovirus, XMRV, in blood cells of patients with chronic fatigue syndrome Science 2009 326 585 589 10.1126/science.1179052 19815723

[B5] Lo SC Pripuzova N Li B Komaroff AL Hung GC Wang R Alter HJ Detection of MLV-related virus gene sequences in blood of patients with chronic fatigue syndrome and healthy blood donors Proc Natl Acad Sci USA 2010 107 15874 15879 10.1073/pnas.1006901107 20798047 PMC2936598

[B6] Hue S Gray ER Gall A Katzourakis A Tan CP Houldcroft CJ McLaren S Pillay D Futreal A Garson JA Pybus OG Kellam P Towers GJ Disease-associated XMRV sequences are consistent with laboratory contamination Retrovirology 2010 7 111 10.1186/1742-4690-7-111 21171979 PMC3018392

[B7] Dong B Kim S Hong S Das Gupta J Malathi K Klein EA Ganem D Derisi JL Chow SA Silverman RH An infectious retrovirus susceptible to an IFN antiviral pathway from human prostate tumors Proc Natl Acad Sci USA 2007 104 1655 1660 10.1073/pnas.0610291104 17234809 PMC1776164

[B8] Kim S Kim N Dong B Boren D Lee SA Das Gupta J Gaughan C Klein EA Lee C Silverman RH Chow SA Integration site preference of xenotropic murine leukemia virus-related virus, a new human retrovirus associated with prostate cancer J Virol 2008 82 9964 9977 10.1128/JVI.01299-08 18684813 PMC2566297

[B9] Kim S Rusmevichientong A Dong B Remenyi R Silverman RH Chow SA Fidelity of target site duplication and sequence preference during integration of xenotropic murine leukemia virus-related virus PLoS One 2010 5 e10255 10.1371/journal.pone.0010255 20421928 PMC2857682

[B10] Bushman F Lewinski M Ciuffi A Barr S Leipzig J Hannenhalli S Hoffmann C Genome-wide analysis of retroviral DNA integration Nat Rev Microbiol 2005 3 848 858 10.1038/nrmicro1263 16175173

